# Identification of novel risk factors for community-acquired *Clostridium difficile* infection using spatial statistics and geographic information system analyses

**DOI:** 10.1371/journal.pone.0176285

**Published:** 2017-05-16

**Authors:** Deverick J. Anderson, Leoncio Flavio Rojas, Shera Watson, Lauren P. Knelson, Sohayla Pruitt, Sarah S. Lewis, Rebekah W. Moehring, Emily E. Sickbert Bennett, David J. Weber, Luke F. Chen, Daniel J. Sexton, the CDC Prevention Epicenters Program

**Affiliations:** 1 Department of Medicine, Duke University, Durham, North Carolina, United States of America; 2 Duke Center for Antimicrobial Stewardship and Infection Prevention, Duke University Durham, North Carolina, United States of America; 3 Breast Oncology Program, Susan F. Smith Center for Women's Cancers at Dana-Farber Cancer Institute, Boston, Massachusetts, United States of America; 4 Duke Health Technology Solutions, Durham, North Carolina, United States of America; 5 Forecast Health, Durham, North Carolina, United States of America; 6 Durham VA Medical Center, Durham, North Carolina, United States of America; 7 Department of Hospital Epidemiology, University of North Carolina at Chapel Hill, Chapel Hill, North Carolina, United States of America; Cleveland Clinic, UNITED STATES

## Abstract

**Background:**

The rate of community-acquired Clostridium difficile infection (CA-CDI) is increasing. While receipt of antibiotics remains an important risk factor for CDI, studies related to acquisition of C. difficile outside of hospitals are lacking. As a result, risk factors for exposure to *C*. *difficile* in community settings have been inadequately studied.

**Main objective:**

To identify novel environmental risk factors for CA-CDI

**Methods:**

We performed a population-based retrospective cohort study of patients with CA-CDI from 1/1/2007 through 12/31/2014 in a 10-county area in central North Carolina. 360 Census Tracts in these 10 counties were used as the demographic Geographic Information System (GIS) base-map. Longitude and latitude (X, Y) coordinates were generated from patient home addresses and overlaid to Census Tracts polygons using ArcGIS; ArcView was used to assess “hot-spots” or clusters of CA-CDI. We then constructed a mixed hierarchical model to identify environmental variables independently associated with increased rates of CA-CDI.

**Results:**

A total of 1,895 unique patients met our criteria for CA-CDI. The mean patient age was 54.5 years; 62% were female and 70% were Caucasian. 402 (21%) patient addresses were located in “hot spots” or clusters of CA-CDI (p<0.001). “Hot spot” census tracts were scattered throughout the 10 counties. After adjusting for clustering and population density, age ≥ 60 years (p = 0.03), race (<0.001), proximity to a livestock farm (0.01), proximity to farming raw materials services (0.02), and proximity to a nursing home (0.04) were independently associated with increased rates of CA-CDI.

**Conclusions:**

Our study is the first to use spatial statistics and mixed models to identify important environmental risk factors for acquisition of *C*. *difficile* and adds to the growing evidence that farm practices may put patients at risk for important drug-resistant infections.

## Introduction

*Clostridium difficile* infection (CDI) leads to adverse patient outcomes [1]. Now the most common pathogen causing healthcare-associated infections (HAI) [2], deaths related to CDI increased 400% between 2000 and 2007 [3]. In fact, the CDC estimates that approximately 500,000 patients have CDI each year in the US, and 29,000 die as a result of the infection [4]. As a result, CDI was recently classified as an “urgent” threat to public health [5].

While the threat from CDI continues to grow, its epidemiology is incompletely understood. More than 50% of CDI cases begin in the community [6], and the rate of community-acquired (CA) CDI is increasing [6, 7]. However, a large proportion of patients who develop CA-CDI lack traditional risk factors such as antimicrobial use or proton pump inhibitor (PPI) exposure [8, 9]. Ultimately, patients admitted to acute care hospitals with CA-CDI in turn impart “CDI pressure” that increases the risk of acquisition of CDI by other vulnerable hospitalized patients that share the same hospital unit [10].

Environmental factors may increase the risk for community acquisition of some traditionally healthcare-associated pathogens, including methicillin-resistant *Staphylococcus aureus* (MRSA) and *C*. *difficile*. For example, the authors of a recent analysis of 1,539 cases of CA-MRSA in a population of 446,480 concluded that proximity to farms that applied swine manure fertilizer was a strong predictor for CA-MRSA skin infection [11]. Similarly, pig farms have recently been associated with CDI, particularly infections caused by ribotype 078 strain [12].

In light of our limited understanding of community reservoirs of *C*. *difficile* and the fact that many patients who develop CA-CDI lack traditional risk factors, new and innovative approaches are needed to determine if additional environmental factors increase the risk for community acquisition of *C*. *difficile*. Methods in spatial statistics including Geographic Information Systems (GIS) are increasingly applied to healthcare investigations and allow researchers to examine outpatient transmission by analyzing the dynamics of spatial configuration of disease over time. We therefore undertook this large, multicenter cohort study using GIS and spatial statistics to identify novel environmental risk factors for CA-CDI.

## Materials and methods

The Duke University Health System (Pro00063169) and University of North Carolina (#15–1712) IRBs approved this research.

### Patient identification

This population-based retrospective cohort study included patients with CDI from two health systems. These systems included two tertiary care hospitals, three community hospitals in the Duke Infection Control Outreach Network [13], and 802 outpatient facilities. Microbiology records were queried to identify all patients with a positive stool test for *C*. *difficile* from January 1, 2007, through December 31, 2014 (hereafter, the “study period”). If a patient had more than one positive test for *C*. *difficile*, only the first test during the study period was included. Patients were defined as having CA-CDI if a positive test was obtained a) at an outpatient clinic or b) during the first 72 hours of a documented hospitalization [14]. Patients who met the above criteria were excluded from our analysis if they had been hospitalized in the prior 12 weeks. Finally, we narrowed our analysis to include only case patients with addresses in a 10-county area surrounding the five study hospitals in an attempt to include the collective catchment area of the study hospitals and health systems in central NC. The population of the 10 study counties was approximately 1.94 million; 1.2 million were located in the two largest population centers: Wake County (Raleigh) and Durham County (Durham).

### Data management and GIS methods

Demographic data for North Carolina were obtained and grouped at Census Tract level using 2010 United Sates Census Bureau Data [15]; 360 Census Tracts in the 10 counties were used as the demographic GIS base-map. We obtained Census Tracts surfaces as measured in square kilometers and converted to square miles [16]. Data related to socioeconomic status were obtained for targeted census tracts from the American Community Survey (ACS) 2008–2012 [17]. Patient home addresses were used to identify longitude and latitude (X, Y) coordinates. The resulting points were overlaid to existing polygons describing the Census Tracts using ArcGIS (version 10.2.2 ESRI, Redlands CA). SAS Data Management Studio was used to USPS verify and standardize each patient address, and then geocode the standardized patient addresses at the rooftop/street level of geography, using USPS and TomTom/TeleAtlas. ArcGIS was used to calculate Euclidean distance (in miles) between case addresses and environmental and geographic variables, as defined by ESRI infrastructure features and MAPINFO Business Points and categorized by standard industrial classification codes (SICCODE) (http://siccode.com/). Environmental variables of interest included proximity to livestock farms, agriculture services, mining services, meat processing facilities, wood mills, sewage treatment facilities, grocery stores, day care facilities, health service facilities such as skilled nursing facilities, hospitals, and dialysis centers, and natural waterways (e.g., rivers, streams, creeks, lakes). SICCODEs used in this study correspond to designations for addresses during 2014

Disease burden within census tracts was initially computed by calculating a rate of cases per thousand per census tract. These unstandardized ratios were then adjusted by age, race, and sex.

### Spatial statistics methods

ArcGIS was used to assess “hot-spots” or clusters of CA-CDI based on a) patient addresses and b) CA-CDI rates. Formal spatial statistical tests using the Getis-Ord statistic were conducted to determine the likelihood that the data configurations were random [18]. The null hypothesis that the geographic distributions of cases were random was rejected if p<0.05. Because patient addresses may indicate clusters due to the tendency of groups with similar risk factors or demographic factors to reside together, the testing of rates was adjusted by age, race, and sex with exact 95% confidence limits to identify correlated clusters of incidence rates. Similarly, we specifically assessed population density to ensure that clusters were not simply reflective of high population density. We also conducted additional tests of temperatures and their monthly average, seasonal average, and variations over time according to geographical location to assess for potential alternative clustered distributions and time-related peaks of CA-CDI. We assessed differences in demographic data between clustered patients and not clustered patients using standard descriptive statistical tests.

### Definitions

Patients and census tracts with Getis-Ord z-scores of ≥1.96 were defined as “clustered;” those with z-scores <1.96 were considered “not clustered”. For the purpose of our analyses, the seven census-based age-groups were collapsed into three categories: <29 years, 30–59 years, 60 years or older. Population densities were calculated for census tracts and categorized as “low” (≤1,729 persons per square mile), “medium” (1,730 to 2,454) and “high” (≥2,455). Definitions of poverty were categorized by Census Tract areas; “low” poverty was defined as 15.6% or less of the population living with an income below the poverty line, “medium” poverty defined as 15.7% to 23.9%, and “high” poverty defined as ≥24% [17].

### Mixed hierarchical model

We constructed a mixed hierarchical model to identify variables independently associated with increased rates of CA-CDI, including distance from important environmental locations. We chose this approach to maximize mixed models’ ability to handle heterogeneous variations associated with spatial heterogeneity and “nuisance” or extra-Poisson variation, which violate the assumption of independence of observations required in standard models. In addition, multilevel mixed models accommodate large numbers of random effects simultaneously with large numbers of fixed effects by evaluating variability as fixed effects at the first level and including random intercepts of individual patients at a second level.

We used the SAS HPMIXED procedure (SAS v9.2, Cary, NC) to create a hierarchical model with multilevel, nested, and spatially clustered observations. Our models included three hierarchical levels: county, census tract, and individual (case patient). More specifically, our modeling approach incorporated polygonal spatial locations (census tracts); individuals were treated as random effects in this model. In contrast, age, sex, race, socioeconomic status, population density, and distance variables were treated as fixed effects. Average monthly temperature and date of *C*. *difficile* test were included to determine if temporal or seasonal trends in CA-CDI incidence were present during the study period. The covariates of the fixed part of the model were tested with Pearson Correlation Coefficients to assess potential collinearity between covariates. Interaction terms between poverty and race as well as population density and patient clusters were evaluated and included in the initial, full model as well.

After developing our initial, full model, we constructed a final, simplified model using backwards elimination of non-significant variables. Fitness statistics confirmed improved fit of the simplified model compared with the full model.

## Results

**Fig 1:**
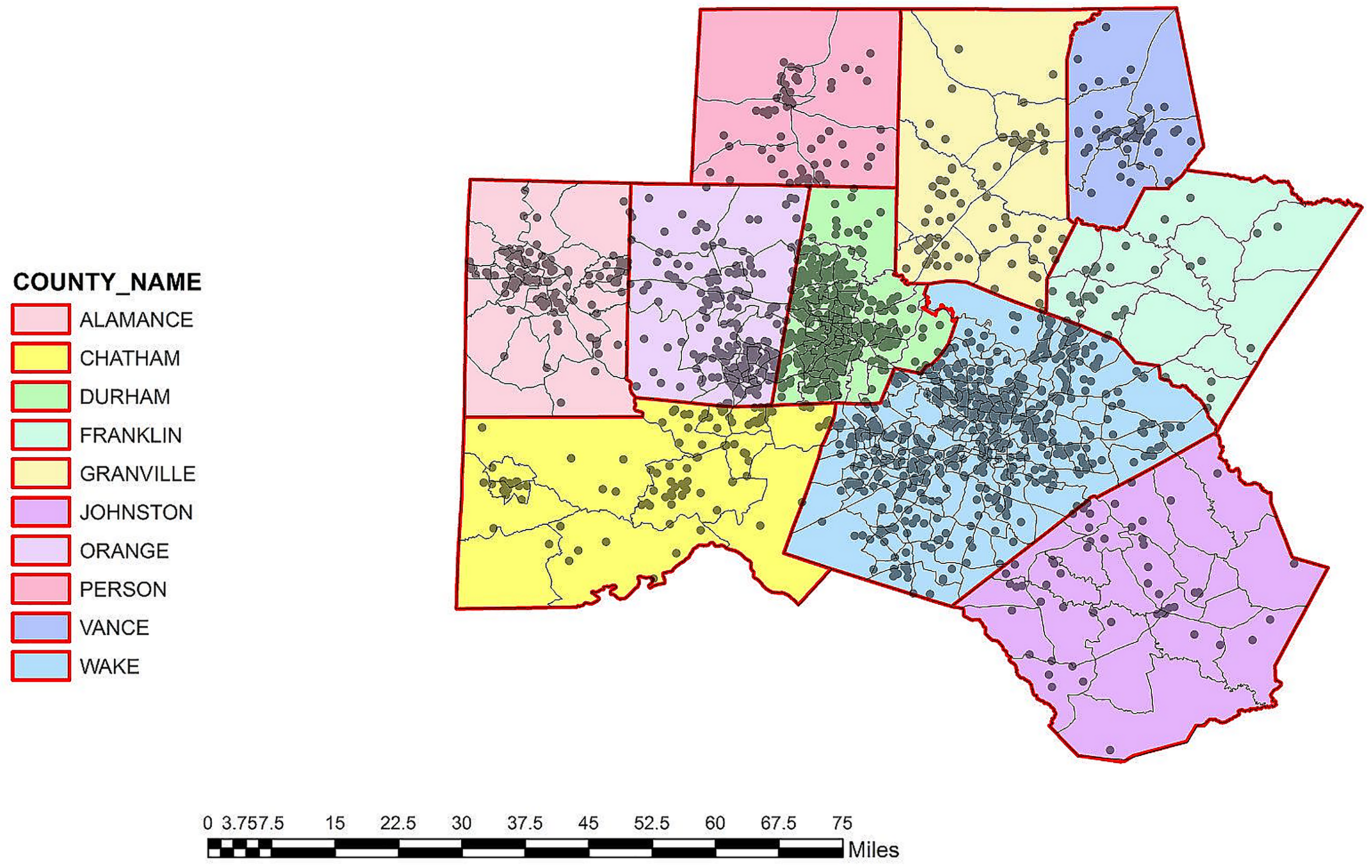
Case location of 1,895 cases of community-associated Clostridium difficile infection in the 10-county study area in central North Carolina. *Census tract size is inversely proportional to population density. Grey dots represent individual cases. North is oriented to the top of the page. MAP SOURCE: Map created using ArcGIS software by Esri using TeleAtlas and US Census data sources.

A total of 8,813 unique patients had a positive test for *C*. *difficile* during the study period; 2,906 patients (33%) met our criteria for CA-CDI, and 1,895 patients with CA-CDI had addresses in the 10-county catchment area for the study hospitals (Fig 1). The mean patient age of our cohort was 54.5; 62% were female and 70% were Caucasian. The population of the 10 study counties was approximately 1.94 million; 1.2 million were located in the two largest population centers: Wake County (Raleigh) and Durham County (Durham).

**Fig 2:**
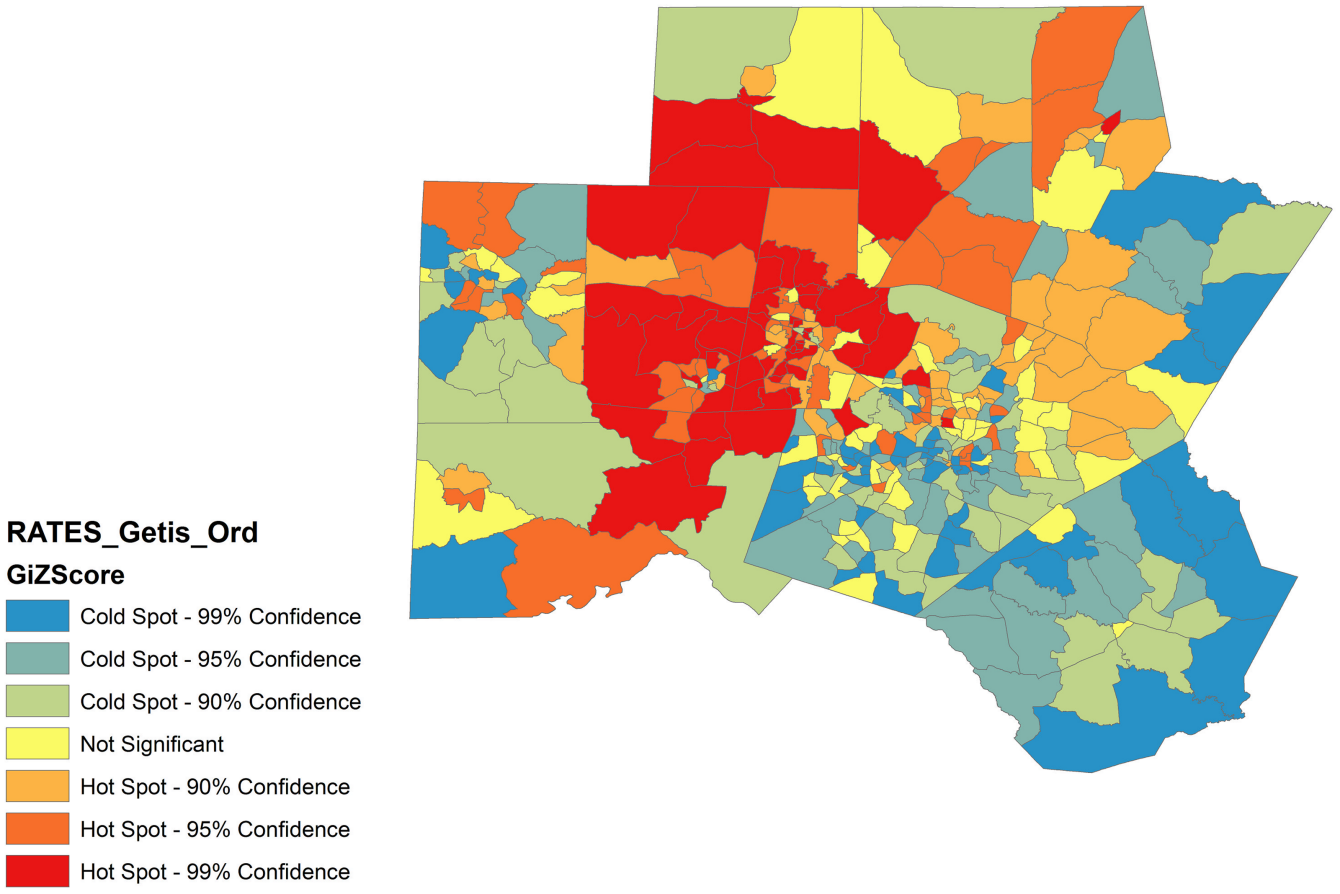
“Hot spots” or clusters of community-acquired CDI in a 10-county area in central North Carolina. North is oriented to the top of the page. MAP SOURCE: Map created using ArcGIS software by Esri using TeleAtlas and US Census data sources.

A total of 402 (21%) patient addresses were located in “hot spots” or clusters of CA-CDI (Getis-Ord p<0.001). Rates of CA-CDI per census tract were also clustered (Getis Ord p<0.001). “Hot spot” census tracts were scattered throughout the 10 counties (Fig 2). Patients with CA-CDI in these clusters were more likely to be Caucasian, younger, and more likely to live in areas of medium or high poverty than patients not residing in clusters (Table 1). Furthermore, CA-CDI “hot spots” were more common in areas of medium population density and areas of medium poverty.

**Table 1:** Comparison of patients with community-acquired *C*. *difficile* infection (CA-CDI) in clusters or “hot spots” of infection versus patients with CA-CDI not located in clusters or hot spots.

	Overall N = 1895 n (%)*	Clustered N = 402 n (%)*	Not Clustered N = 1493 n (%)*	p-value
Age—mean ± SD	54.5 ± 21.7	52.8 ± 24.2	54.9 ± 23.5	0.05
≤29 years	310 (16)	77 (19)	233 (16)	
30 to 59 years	689 (36)	147 (37)	542 (36)	
≥60 years	896 (47)	178 (44)	718 (48)	
Female sex	1183 (62)	246 (61)	937 (63)	0.61
Race				0.001
Caucasian	1329 (70)	305 (76)	1024 (69)	
African American	430 (23)	64 (16)	366 (25)	
Other	136 (7)	33 (8)	103 (7)	
Population density				0.02
High	282 (15)	53 (14)	229 (15)	
Medium	390 (21)	102 (25)	288 (19)	
Low	1223 (65)	247 (61)	976 (65)	
Socioeconomic status				0.04
High poverty	301 (16)	68 (17)	233 (16)	
Medium poverty	382 (20)	97 (24)	285 (19)	
Low poverty	1212 (64)	237 (60)	975 (65)	

* percentages may not add up 100 due to rounding

After adjusting for clustering and population density, two patient-specific variables were independently associated with increased rates of CA-CDI: age ≥ 60 years and race. Several environmental variables were associated with rates of CA-CDI. Increasing proximity to a livestock farm, increasing proximity to farming raw materials services, and increasing proximity to a nursing home were associated with increasing rates of CA-CDI (Table 2). In addition, increasing distances from meat processing plants, hospitals, and wood mills were each independently associated with increased rates of CA-CDI in our exploratory model. No linear temporal or seasonal/temperature trends in rates were observed in our models.

**Table 2:** Fixed effects variables in the final, reduced hierarchical model* to determine factors independently associated with community-associated *Clostridium difficile* infection (CA-CDI).

Variables	Model Estimate	SE	p-value
Age	0.086	0.041	0.03
Race	2.26	0.25	<0.001
Population density category	-0.29	0.14	0.04
Proximity to**			
Livestock farm	-0.021	0.009	0.01
Nursing home	-0.019	0.009	0.04
Farm raw materials services	-0.011	0.005	0.02
Meat processing plant	0.027	0.007	<0.001
Hospital	0.041	0.010	<0.001
Wood mill	0.041	0.013	0.001

*Model controlled for potential confounding from socioeconomic status, proximity to mining, and interactions between a) socioeconomic status and race and b) cluster and population density. Wood mill was included in the analysis for check for model validity. Wood mill and farming locations serve as inverse geographic variables. That is, locations close to farming locations are further away from wood mill locations and vice-versa. As expected, proximity to wood mill was significant, but inverse to the relationship observed for livestock farm.

**Proximity variables based on SICCODES, negative values for estimates implies correlation with smaller values (i.e., closer proximity to the environmental location).

SE—Standard Error

## Discussion

Our large, multicenter study is the first to use GIS and spatial statistics to identify both specific geographic clusters and novel environmental risk factors for *C*. *difficile* infection (CDI) acquired in the community. Proximity to livestock farms and proximity to facilities that handle raw farming materials were independently associated with increasing rates of CA-CDI. Our model also supports findings from prior studies by showing that age greater than 60 years and nursing homes were independently associated with increased rates of CA-CDI [19].

Additional studies are needed to understand why proximity to farms and farming services are associated with increased rates of CA-CDI. The prevailing model for the pathogenesis of CDI requires a perturbation of the gut flora (e.g., medication or procedure) and exposure to/acquisition of *C*. *difficile* [20]. Prior studies of the risk factors for CA-CDI have primarily focused on the factors that alter gut flora and render patients susceptible, including antimicrobial therapy and exposure to proton pump inhibitors [21–23]. In contrast, few studies have investigated community reservoirs for exposure to and acquisition of *C*. *difficile*. These studies have identified exposure to household contacts with *C*. *difficile* [24] and exposure to children less than one1 year of age in the household as risk factors for CA-CDI [8, 25]. Other investigators have identified *C*. *difficile* in retail food, livestock, domestic animals, and wild animals [26–31], but no prior studies have demonstrated that proximity to or interaction with these potential animal reservoirs is a risk for subsequent human infection.

Despite John Snow’s original use of geographic mapping to identify an important source for an infectious disease over 150 years ago, the strategy has infrequently been employed to investigate environmental risk factors and healthcare-associated and/or multidrug-resistant pathogens. GIS and spatial statistics were recently used to evaluate risk factors for MRSA infection among 867,254 people in a three-borough catchment area in London [32]. The risk of CA-MRSA was increased in areas with important socioeconomic factors such as overcrowding, homelessness, low income, and recent immigration. Geographic areas adjacent to these high-risk areas were also at increased risk, confirming the impact of geographic proximity to high risk areas. To our knowledge, however, no prior studies have used GIS and spatial statistics to investigate risk factors for *C*. *difficile* acquisition and infection in the community. Chitnis et al. recently summarized 984 patients with CA-CDI identified through the CDC’s Emerging Infection Program. Similar to our study, the median age of patients in this cohort was 51 and the majority were female [8]. A total of 345 (36%) patients had no antibiotic exposure and 177 (18%) had no known healthcare exposure. Occupational exposure to animals was not found to be a risk factor for CA-CDI, though only 22 (2%) patients had this exposure in the entire cohort. Other environmental or geographic factors were not evaluated in this investigation.

Proximity to farms has also previously been show to put patients at risk for acquisition of other multidrug-resistant organisms such as MRSA [33–35]. This increased risk in populations that live close to livestock operations and farms may be related to practices used in modern farming, including the application of swine manure to fields. Aerosolized MRSA isolates generated on farms can be identified in the air up to 150m downwind and in the soil up to 300m downwind [36, 37]. In fact, proximity to farms that apply swine manure to crop fields and livestock operations is associated with a 1.4-fold increase in CA-MRSA infection, a 1.3-fold increase in HA-MRSA, and a 1.4-fold increase in skin and soft tissue infection [11]. Of note, this exposure also led to a 30% increase in risk of HA-MRSA, implying that patients exposed to MRSA from farm practices can import MRSA into hospitals.

By our review, proximity to livestock farms has not previously been described as a risk factor for CDI. We believe this association is plausible. Multiple studies have documented the presence of *C*. *difficile* in the farm environment and farm workers, and identified common *C*. *difficile* strains and clones in both livestock and humans. First, 80% of the antimicrobials used in the US are used in livestock [38], which likely increases selection for *C*. *difficile*. Second, pathogenic *C*. *difficile* isolates, most notably ribotypes 078 and 027, have been isolated on farms and from farm animals such as pigs. For example, Hopman et al. evaluated 71 newborn piglets and observed that they were routinely colonized with *C*. *difficile* ribotype 078 within 48 hours of birth. This strain of *C*. *difficile* was also cultured from soil and air samples from pig farms; 20 of 21 isolates evaluated were clonal by multiple locus variable number tandem repeat analysis (MLVA) [39]. Ribotype 078 is the most common circulating strain among both pigs and humans in Spain [40], and the third most common strain in humans in Europe [41]. Keessen et al investigated 32 hog farms in the Netherlands and identified *C*. *difficile* ribotype 078 in pig manure in all farms [12]. Humans were exposed to and colonized by the same strains as the pigs in this study; more specifically, 25% of people with direct interaction with pigs on the farms had *C*. *difficile* colonization of stool. Pig and human *C*. *difficile* isolates were identical by MLVA in 13 of 15 farms evaluated. These studies, however, did not evaluate for CA-CDI in the general population living near these farms.

Our study has limitations. First, this analysis was performed as a hypothesis-generating exercise that needs further validation. Though plausible, our findings are inferential and do not demonstrate clear causality. Second, our dataset did not include specific information regarding long term care facility (LTCF)-associated CDI. However, our final model found risk factors independently associated with increasing rates of CA-CDI despite including proximity to nursing home, which we believe should adequately account for LTCF-associated CDI. Third, all hospitals transitioned to more sensitive PCR testing for *C*. *difficile* during the study period. We tested for temporal trends in our models, however, and concluded that year did not impact the fixed effects estimates in our models. Fourth, our study was performed in North Carolina, the second largest pig producing state in the US. While findings from our models specifically related to proximity to livestock farms may have limited generalizability, we believe our results are a useful demonstration of the potential utility of GIS methods in epidemiological studies. In addition, risk related to proximity may not be specifically related to the type of farm but the practices performed at the farm (e.g., spraying of manure). Next, our models were built on assumptions such as a stable census (measured in 2010) and stable SICCODEs. In reality, we suspect that population changes occurred and businesses changed during the seven-year study period. We believe the error introduced by these changes, however, would be random error and, thus, believe that the use of these assumptions in our model was reasonable. In addition, we did not check residual spatial variation in our regression residuals. Finally, our analysis included only limited patient-specific variables and did not include antibiotic exposure for the patient cohort. However, the risk factors that we identified in our analysis may reflect potential risk factors for *C*. *difficile* exposure and acquisition, rather than risk factors for perturbation of colonic flora.

In summary, our study is the first to use GIS, spatial statistics, and mixed models to investigate potential environmental risk factors for acquisition of *C*. *difficile*. Using this novel approach, we found that proximity to livestock farms, proximity to facilities that handle raw farming materials, age, and nursing homes were risk factors for CA-CDI. Our data adds to the growing evidence that farms and farming practices, such as regular use of antibiotics in livestock and use of manure, may increase the risk of disease among people living near these facilities. Subsequent molecular studies will be required to more definitively demonstrate causality. Further investigation and confirmation, however, is important, as increasing rates of CA-CDI have direct impact on the spread of CDI within hospitals [42]. In light of increasing information suggesting that CDI spread in the hospital originates from community reservoirs [43], more studies like ours are needed.

## APPENDIX 1. Detailed hierarchical model

### Variables and variable definitions

Subscripts: *k* ^th^ observation from *j* ^th^ patient in the *i* ^th^ area (polygons, catchment areas)

### Fixed effects variables

$$\begin{array}{ll} {\text{Y}}_{\text{ijk}}=\;\mu \;+ & \beta_{1}\left({\text{GetOrd}}_{\text{ijk}}\right){\;+\;\beta }_{\text{2}}{}_{\;}{(\text{STD}\_\text{COUNTY}}_{\text{ijk}}{)\;+\;\beta }_{\text{3}}{}_{\;}{\text{(agecat2}}_{\text{ijk}}{)+\;\beta }_{\text{4}}{\text{(race4}}_{\text{ijk}}\text{)+}\\ & \beta_{\text{5}}{}_{\;}{\text{(pctpovcat}}_{\text{ijk}}{)+\;\beta }_{6}{\text{(popdenscat}}_{\text{ijk}})+\\ & \beta_{\text{7}}{}_{\;}{(\text{livestock}\_\text{farm}}_{\text{ijk}}{)+\;\beta }_{\text{8}}{\text{(mining}}_{\text{ijk}}{)+\;\beta }_{\text{9}}{}_{\;}{(\text{meat}\_\text{processing}}_{\text{ijk}}\text{)+}\\ & \beta_{\text{10}}{}_{\;}{(\text{wood}\_\text{mills}}_{\text{ijk}}{)+\;\beta }_{\text{11}}{}_{\;}{(\text{farm}\_\text{raw}\_\text{materials}}_{\text{ijk}}{)+\;\beta }_{\text{12}}{}_{\;}{(\text{nursing}\_\text{home}}_{\text{ijk}}\text{)+}\\ & \beta_{\text{13}}{\text{(hospital}}_{\text{ijk}}{)+\;\beta }_{\text{4}}{\text{(race4}}_{\text{ijk}}{)*\beta }_{\text{5}}{}_{\;}{\text{(pctpovcat}}_{\text{ijk}})+\;\beta 1\left({\text{GetOrd}}_{\text{ijk}}\right){*\beta }_{6}{}_{\;}{\text{(popdenscat}}_{\text{ijk}})\;+\;\\ & {\text{a}}_{\text{j}}\left(\text{Patient}\right){\;+\text{ b}}_{\text{ij}}\;+\;e_{\text{ijk}} \end{array}$$

μ = Intercept, Overall mean

β_1_ = GetOrd (Hot-spot indicator, 1 = clustered 0 = not clustered)

β_2_ = Counties (catchment area counties 1, 2, 3… 8, 9, 10)

β_3_ = Age categories

β_4_ = Race categories (“Whites”, “Blacks/African-American”, “Other”)

β_5_ = Poverty in percentages of individuals living below poverty line (“high”, “medium”, “low”)

β_6_ = Population density in number of persons per Square Miles (“high”, “medium”, “low”)

β_7_ = Livestock farm distance from patient address in Square miles

β_8_ = Mining site distance from patient address in Square miles

β_9_ = Meat processing plants distance from patient address in Square miles

β_10_ = Wood mills distance from patient address in Square miles

β_11_ = Farm raw materials processing site distance from patient address in Square miles

β_12_ = Nursing-home facility distance from patient address in Square miles

β_13_ = Hospital distance from patient address in Square miles

β_4_ (race4_ijk_)* β_5_ (pctpovcat_ijk_) = Interactions of race by poverty

β_1_ (GetOrd_ijk_)* β_6_ (popdenscat_ijk_) = Interactions of hot-spots by population density

a_j_ = Patients (Random intercepts)

b_ij_ = Patients nested to catchment areas

*e*_ijk_ = Random residual

## Supporting information

S1 File Image copyright information. Copyright details and information regarding Figs 1 and 2.
(PDF)
