# Supplementary material for: Identification of novel risk factors for community-acquired Clostridium difficile infection using spatial statistics and geographic information system analyses
Source: PLoS One. 2017 May 16;12(5):e0176285. doi: 10.1371/journal.pone.0176285 (PMC5433765; doi:10.1371/journal.pone.0176285)
Supplement: S1 File — Copyright details and information regarding Figs 1 and 2. (PDF) [file pone.0176285.s001.pdf]

Sign in

Industries Products Support & Services About Community

ARCGIS BLOG

Search posts Search

Using and citing Esri data

Esri Home

by abuckley on December 3, 2010

13 9 11.9K

By Aileen Buckley, Mapping Center Lead

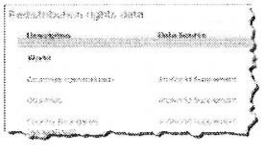

Many of you have asked us about how to cite Esri or other data that you use on your maps. In this blog entry, we describe how to do that properly. As an example, we will use the data that you can access in the Esri Data & Maps Media Kit. The Esri Data & Maps Media Kit is a wonderful collection of data that is provided with the software under license. The data are listed on what is referred to as the "Redistribution Rights Matrix". This Matrix is also located on our website at this link for the most current version of Data & Maps.

When you use the data, you should cross check each data layer and consult the Matrix for information on the Redistribution Rights. If you click on the link above and look up the data layers you are using under the appropriate category (World, USA, etc.) you will find a redistribution code on the right hand side. The definitions for the codes are located in the subsequent pages (the FAQ's section) of that website link. Simply scroll down to find the definitions. You should be able to find the redistribution rights for any data layer you are using.

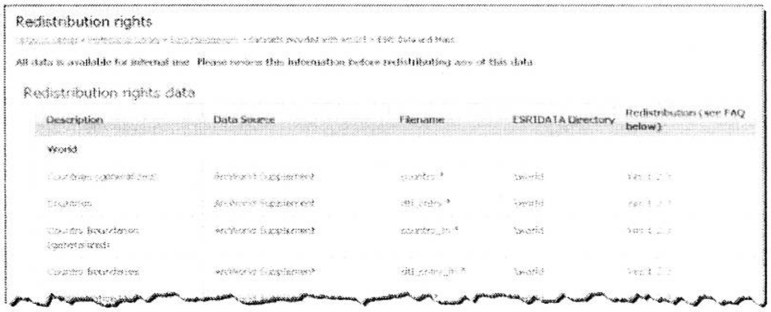

Redistribution rights

All data is available for internal use. Please review this information before redistributing any of this data.

Redistribution rights data

Description	Data Source	Filename	ESRIDATA Directory	Redistribution (see FAQ below)
World				
[illegible]	ArcWorld Supplement	[illegible]	\world	[illegible]
[illegible]	ArcWorld Supplement	[illegible]	\world	[illegible]
[illegible]	ArcWorld Supplement	[illegible]	\world	[illegible]
[illegible]	ArcWorld Supplement	[illegible]	\world	[illegible]

For example, if you are using the data in a hard copy, static format, you will need a "Yes 1" at a minimum or a "Yes 4" for the data layers you are using. Because this Matrix is in place for the data in the media kit, Esri does not provide formal permission grants for requests you may send us to use these data. It is up to you, the end user, to consult the Matrix and confirm that the type of use you propose is in fact permitted by the data vendor(s). Once the end user confirms that he or she has the proper rights to use the data as they have proposed, all that is required is that the data vendor be sourced for the use of the data.

The data vendor information is conveniently located on the Matrix in the second column. For example, under Canada, the shapefile "**province.***" is a "Yes 1, 2" for Redistribution. You would then refer to the column "Data Source" and find that this data was provided under license by **DMTI Spatial Inc**. So, following this example, for the Canada layer "province.*" your attribution for the map created using this

data should read "**Source: DMTI Spatial Inc**." This should be done for each data layer being used to create a map.

With regard to a statement on the map or in the publication (e.g., book, article) in which the map appears, one way you could reference the use of Esri software to create your maps is a statement such as:

"**Maps throughout this book were created using ArcGIS® software by Esri. ArcGIS® and ArcMap™ are the intellectual property of Esri and are used herein under license. Copyright © Esri. All rights reserved. For more information about Esri® software, please visit** www.esri.com."

Thanks to Lisa Horn for her help with this blog entry.

This entry was posted in Mapping and tagged citing, Map Data, Publishing, using. Bookmark the permalink.

2 Comments

mheberger *says:*

The suggestion at the end of this post seems particularly heavy-handed. Following this suggestion, I'd have to write:

Text was prepared in Microsoft Word, copyright Microsoft Corporation... Tabular data compiled in MS Excel... Images cropped and resized in IrfanView... ad nauseum.

December 23, 2010 at 6:15 pm Log in to Reply

abuckley *says:*

Good point! I edited the sentence to make it less "heavy handed"! Hope this helps!

February 18, 2011 at 8:58 pm Log in to Reply

Follow Us

Understanding GIS
What is GIS?
Map Book Gallery
Video Library

Company Information
About Esri
Careers
Esri Insider Blog
Esri User Conference
Trust ArcGIS

Services
Professional Services
Project Services
Implementation Services
Premium Support Services

Partners
Learn About Partners
Partner Directory
Hardware Promotions

Special Programs
Esri Special Programs
Nonprofit
Education
Disaster Response
ArcGIS for Personal Use
Conservation

Contact Us | Privacy | Legal | Site Map

Redistribution rights

Resource Center » Professional Library » Data Management » Datasets provided with ArcGIS » ESRI Data and Maps

All data is available for internal use. Please review this information before redistributing any of this data.

Redistribution rights data

Description	Data Source	Filename	ESRIDATA Directory	Redistribution (see FAQ below)
**World**				
Countries (generalized)	ArcWorld Supplement	country.*	\world	Yes 1,2,3
Countries	ArcWorld Supplement	dtl_cntry.*	\world	Yes 1,2,3
Country Boundaries (generalized)	ArcWorld Supplement	country_ln.*	\world	Yes 1,2,3
Country Boundaries	ArcWorld Supplement	dtl_cntry_ln.*	\world	Yes 1,2,3
Administrative Units	ArcWorld Supplement	admin.*	\world	Yes 1,2,3
Administrative Boundaries	ArcWorld Supplement	admin_ln.*	\world	Yes 1,2,3
Airports	DeLorme	airports.*	\world	Yes 1,2,3
Contours	DeLorme	contours.*	\world	Yes 1,2,3
Roads	DeLorme	roads.*	\world	Yes 1,2,3
Railroads	DeLorme	railroads.*	\world	Yes 1,2,3
Continents	ArcWorld Supplement	continent.*	\world	Yes 1,2,3
Regions	ArcWorld Supplement	region.*	\world	Yes 1,2,3
Census IPC Demographics (table)	US Census International Division, CIA Factbook	cntry_ipc.*	\world	Yes 1,2,3
Country Memberships of Political Organizations (table)	CIA Factbook	pol_org.*	\world	Yes 4
Cities	ArcWorld	cities.*	\world	Yes 1,2,3
Populated Places	DeLorme	pop_places.*	\world	Yes 1,2,3
Urban Areas	DeLorme	urban_areas.*	\world	Yes 1,2,3
Gazetteer	DCW	gaz.*	\world	Yes 1,2,3
Linear Water	DeLorme	hydrolines.*	\world	Yes 1,2,3

Water Bodies	DeLorme	hydropolys.*	\world	Yes 1,2,3
Drainage Systems, Lakes, and Rivers	ArcWorld	lakes.*, rivers.*, drainage.*	\world	Yes 1,2,3
World Wildlife Fund Terrestrial Ecoregions	World Wildlife Fund, DCW	wwf_terr.*	\world	No
World Wildlife Fund Marine Ecoregions	World Wildlife Fund, DCW	wwf_mar.*	\world	No
UTM Zones	ArcWorld Supplement	utmzone.*	\world	Yes 1,2,3
Time Zones	ESRI	timezone.*	\world	Yes 1,2,3
Latitude and Longitude Grids	ESRI	latlong.*	\world	Yes 1,2,3
Named Latitudes and Longitudes	ESRI	geogrid.*	\world	Yes 1,2,3
Map Background	ESRI	world30.*	\world	Yes 1,2,3
**StreetMap North America**				
States and Provinces	Tele Atlas	states.*	\streetmap_na	Yes 1,2
State and Province Boundaries	Tele Atlas	stborder.*	\streetmap_na	Yes 1,2
US Counties	Tele Atlas	counties.*	\streetmap_na	Yes 1,2
US County Boundaries	Tele Atlas	countybnd.*	\streetmap_na	Yes 1,2
City Points	Tele Atlas	cities.*	\streetmap_na	Yes 1,2
Major Cities	Tele Atlas	citiesmjr.*	\streetmap_na	Yes 1,2
City Areas	Tele Atlas	citylim.*	\streetmap_na	Yes 1,2
Postal Points	Tele Atlas	zipcentr.*	\streetmap_na	Yes 1,2
Postal Areas	Tele Atlas	zip5.*	\streetmap_na	Yes 1,2
Maneuvers	Tele Atlas	maneuver.*	\streetmap_na	Yes 1
Highway Exits	Tele Atlas	exits.*	\streetmap_na	Yes 1
Connector Roads	Tele Atlas	connectors.*	\streetmap_na	Yes 1,2
Major Roads	Tele Atlas	mroads.*	\streetmap_na	Yes 1,2
Highways	Tele Atlas	highways.*	\streetmap_na	Yes 1,2
Interstate Highways	Tele Atlas	interstates.*	\streetmap_na	Yes 1,2
Railroads	Tele Atlas	railroads.*	\streetmap_na	Yes 1,2
Lakes	Tele Atlas	lakes_na.*	\streetmap_na	Yes 1,2
Rivers	Tele Atlas	rivers.*	\streetmap_na	Yes 1,2
Water Polygons	Tele Atlas	waterp.*	\streetmap_na	Yes 1,2

Major Water Polygons	Tele Atlas	waterpmjr.*	\streetmap_na	Yes 1,2
Airports	Tele Atlas	airports.*	\streetmap_na	Yes 1,2
Institutions	Tele Atlas	institutions.*	\streetmap_na	Yes 1,2
Large Area Landmarks	Tele Atlas	landmarks.*	\streetmap_na	Yes 1,2
Parks	Tele Atlas	parks.*	\streetmap_na	Yes 1,2
Recreation Areas	Tele Atlas	recarea.*	\streetmap_na	Yes 1,2
Transportation Terminals	Tele Atlas	transterm.*	\streetmap_na	Yes 1,2
Retail Centers	Tele Atlas	retail_center.*	\streetmap_na	Yes 1,2
Cartographic Streets	Tele Atlas	streetscarto.*	\streetmap_na	Yes 1
Detailed Streets	Tele Atlas	streets.*	\streetmap_na	Yes 1
**United States**				
States and Counties (generalized)	ArcUSA, U.S. Census, ESRI (Pop2010 fields)	states.*, counties.*	\usa\census	Yes 1,2,3
States and Counties	ESRI, derived from Tele Atlas, U.S. Census, ESRI (Pop2010 fields)	dtl_st.*, dtl_cnty.*	\usa\census	Tele Atlas—Yes 1,2 U.S. Census —Yes 1,2,3 ESRI—Yes 1,2,3
State and County Boundaries	ESRI, derived from Tele Atlas	dtl_st_ln.*, dtl_cnty_ln.*	\usa\census	Tele Atlas—Yes 1,2
County Population Estimates (tables)	U.S. Census, Federal State Cooperative Program for Population Estimates	popestmt90.*, popestmt00.*	\usa\census	Yes 4
Census Tracts	Tele Atlas, U.S. Census, ESRI (Pop2010 fields)	tracts.*	\usa\census	Tele Atlas—Yes 1,2 U.S. Census —Yes 1,2,3 ESRI—Yes 1,2,3
Census Feature Class Codes (table)	U.S. Census	cfcc.*	\usa\census	Yes 4
110th Congressional Districts	ESRI	cd110.*	\usa\census	Yes 1,2,3
111th Congressional Districts	ESRI	cd111.*	\usa\census	Yes 1,2,3
Cities	U.S. Census	cities.*	\usa\census	Yes 4
Populated Place Points	U.S. Census	places.*	\usa\census	Yes 4

Populated Place Areas	Tele Atlas, U.S. Census	placeply.*	\usa\census	Yes 1,2
Core-based Statistical Areas	Tele Atlas	cbsa.*	\usa\census	Yes 1,2
Major Roads	Tele Atlas	mjrrds.*	\usa\trans	Yes 1,2
Highways	ESRI	highways.*	\usa\trans	Yes 1,2,3
Major Highways	ESRI	mjr_hwys.*	\usa\trans	Yes 1,2,3
National Transportation Atlas—Interstate Highways	U.S. Bureau of Transportation Statistics	intrstat.*	\usa\trans	Yes 4
National Transportation Atlas—Railroads	U.S. Bureau of Transportation Statistics	rail100k.*	\usa\trans	Yes 4
Census Urbanized Areas	U.S. Census	urban.*	\usa\census	Yes 4
Major Parks	National Park Service, ArcUSA, Tele Atlas	parks.*	\usa\landmarks	Yes 1,2
Drainage Systems, Lakes, and Rivers (generalized)	ArcWorld	lakes.*, rivers.*, drainage.*	\usa\hydro	Yes 1,2,3
Telephone Area Code Boundaries	Tele Atlas	areacode.*	\usa\census	Yes 1,2
ZIP Code Points	Tele Atlas, ESRI (Pop2010 fields)	zip_usa.*	\usa\census	Tele Atlas—Yes 1,2 ESRI—Yes 1,2,3 U.S. Census —Yes 1,2,3
ZIP Code Areas (Five-Digit)	Tele Atlas, ESRI (Pop2010 fields)	zip_poly.*	\usa\census	Tele Atlas—Yes 1,2 ESRI—Yes 1,2,3 U.S. Census —Yes 1,2,3
ZIP Code Areas (Three-Digit)	ESRI, derived from Tele Atlas, ESRI (Pop2010 fields)	zip3.*	\usa\census	Tele Atlas—Yes 1,2 ESRI—Yes 1,2,3 U.S. Census —Yes 1,2,3
National Atlas—Airports	National Atlas of the United States	airports.*	\usa\trans	Yes 4
National Atlas—Cities	National Atlas of the United States	cities_dtl.*	\usa\census	Yes 4
		urban_dtl.*	\usa\census	Yes 4

National Atlas—Urban Areas	National Atlas of the United States			
National Atlas—Federal and Indian Land Areas	National Atlas of the United States	fedlandp.*	\usa\other	Yes 4
National Atlas—Federal Land Lines	National Atlas of the United States, USGS	fedlandl.*	\usa\other	Yes 4
National Atlas—Water Feature Areas and Lines	National Atlas of the United States, USGS	hydroply.*, hydroln.*	\usa\hydro	Yes 4
National Atlas—Public Land Survey	National Atlas of the United States, USGS	publdsur.*	\usa\other	Yes 4
National Atlas—Historic Earthquakes	National Atlas of the United States, USGS	quakehis.*	\usa\landmarks	Yes 4
National Atlas—Volcanoes	Smithsonian Institution, Global Volcanism Program, National Atlas of the United States	volcano.*	\usa\landmarks	Yes 4
Airports	Tele Atlas	airportp.*	\usa\trans	Yes 1,2
Institutions	Tele Atlas	institut.*	\usa\landmarks	Yes 1,2
Large Area Landmarks	Tele Atlas	lalndmrk.*	\usa\landmarks	Yes 1,2
Parks	Tele Atlas	park_dtl.*	\usa\landmarks	Yes 1,2
Recreation Areas	Tele Atlas	recareas.*	\usa\landmarks	Yes 1,2
Transportation Terminals	Tele Atlas	tranterm.*	\usa\trans	Yes 1,2
Geographic Names Information System—Buildings	USGS—GNIS	gblding.*	\usa\landmarks	Yes 4
Geographic Names Information System—Cemeteries	USGS—GNIS	gcemetry.*	\usa\landmarks	Yes 4
Geographic Names Information System—Churches	USGS—GNIS	gchurch.*	\usa\landmarks	Yes 4
Geographic Names Information System—Golf Locales	USGS—GNIS	ggolf.*	\usa\landmarks	Yes 4
	USGS—GNIS	glocale.*	\usa\landmarks	Yes 4

Geographic Names Information System—Locales				
Geographic Names Information System—Populated Places	USGS—GNIS	gppl.*	\usa\landmarks	Yes 4
Geographic Names Information System—Hospitals	USGS—GNIS	ghospitl.*	\usa\landmarks	Yes 4
Geographic Names Information System—Schools	USGS—GNIS	gschools.*	\usa\landmarks	Yes 4
Geographic Names Information System—Summits	USGS—GNIS	gsummit.*	\usa\landmarks	Yes 4
State Plane Zones (NAD 1927, NAD 1983)	NOAA, USGS, ESRI	spcszn27.*, spcszn83.*	\usa\other	Yes 1,2,3
USGS Topographic Quadrangle Series Indexes—1:24,000, 1:100,000, 1:250,000	ArcUSA	topoq24.*, topoq100.*, topoq250.*	\usa\other	Yes 1,2,3
Census Block Groups	Tele Atlas, U.S. Census, ESRI (Pop2010 fields)	blkgrp.*	\usa\census	Tele Atlas—Yes 1,2 U.S. Census —Yes 1,2,3 ESRI—Yes 1,2,3
Census Block Centroid Populations	U.S. Census	blockpop.*	\usa\census	Yes 1,2
Rivers and Streams	USGS, ESRI	dtl_riv.*	\usa\hydro	Yes 1,2,3
Water Bodies	USGS, ESRI	dtl_wat.*	\usa\hydro	Yes 1,2,3
**Europe**				
Europe Demographic - NUTS 0 Demographics	Michael Bauer Research GmbH, EuroGeographics	nuts0.*	\europe\demography	Yes 1
Europe Demographic - NUTS 1 Demographics	Michael Bauer Research GmbH, EuroGeographics	nuts1.*	\europe\demography	Yes 1
Europe Demographic - NUTS 2 Demographics	Michael Bauer Research GmbH, EuroGeographics	nuts2.*	\europe\demography	Yes 1
Europe Demographic -	Michael Bauer Research GmbH, EuroGeographics	nuts3.*	\europe\demography	Yes 1

NUTS 3 Demographics				

This table describes what the redistribution rights are for each Data & Maps layer

Frequently asked questions (FAQs)

Q: Is all of the sample data provided on the ESRI Data & Maps disks freely redistributable?

A: No. Much of the sample data is provided by various, third party data vendors under license to ESRI for inclusion on ESRI Data & Maps disks for use with ESRI software.

Q: What does the last column "Redistribution" on the Redistribution Rights matrix with its "Yes" or "No" answers mean?

A: Each data vendor has its own data licensing policies and may grant varying redistribution rights to end users. Please consult the Redistribution Rights Codes below to determine the redistribution rights for a certain sample data file provided on ESRI Data & Maps disks. As used herein, "geodata" shall mean any digital dataset consisting of geographic data coordinates and associated attributes.

- No—Internal Use Only. No redistribution rights are granted by the data vendor, and the data is for the end user's own internal use only.
- Yes 1—Redistribution rights are granted by the data vendor for hardcopy renditions or static, electronic map images (for example, .gif, .jpeg) that are plotted, printed, or publicly displayed with proper metadata and source/copyright attribution to the respective data vendor/vendors.
- Yes 2—Geodata is redistributable with a value-added software application developed by ESRI Business Partners on a royalty-free basis with proper metadata and source/copyright attribution to the respective data vendor/vendors.
- Yes 3—Geodata is redistributable without a value-added software application (that is, adding the sample data to an existing, [non]commercial dataset for redistribution) with proper metadata and source/copyright attribution to the respective data vendor/vendors.
- Yes 4—Public domain data from the U.S. government is freely redistributable with proper metadata and source attribution.

Q: Are there any legal terms and conditions I need to be aware of under this license to use the sample data provided on ESRI Data & Maps disks?

A: Yes. The Terms and Conditions below apply to all the sample datasets provided on the ESRI Data & Maps disks.

High Risk Activities: (a) The Software, Data, and Documentation are not fault-tolerant and are not designed, manufactured, or intended for use or resale for insurance underwriting or with critical health and safety or online control equipment in hazardous environments that require fail-safe performance, such as in the operation of nuclear facilities, aircraft navigation, or communication systems, air traffic control, emergency response, terrorism prevention or response, life support, or weapons systems ("High Risk Activities"). ESRI SPECIFICALLY DISCLAIMS ANY EXPRESS OR IMPLIED WARRANTY OF FITNESS FOR HIGH RISK ACTIVITIES.

(b) To the extent permitted by law, Licensee agrees to indemnify, defend, and hold ESRI, its officers, directors, employees, agents, subcontractors, licensors, successors, and assigns harmless from and against any and all liability, losses, claims, expenses (including attorneys' fees), demands, or damages of any kind, including direct, indirect, special, punitive, incidental, or consequential damages, arising out of or in any way connected with Licensee's use or permitting the use by others of the Software, Data, and vendor's hardware for High Risk

Activities. Delivery of the Software, Data, and vendor's hardware does not constitute a waiver of the rights and obligations set forth in this Article.

Proprietary Rights and Copyright: Licensee acknowledges that the Data and Related Materials contain proprietary and confidential property of ESRI and its licensor/licensors. The Data and Related Materials are owned by ESRI and its licensor/licensors and are protected by United States copyright laws and applicable international copyright treaties and/or conventions.

Limited Warranty and Disclaimer: ESRI warrants that the media upon which the Data and Related Materials are provided will be free from defects in materials and workmanship under normal use and service for a period of ninety (90) days from the date of receipt.

THE DATA AND RELATED MATERIALS ARE EXCLUDED FROM THE LIMITED WARRANTY, AND THE LICENSEE EXPRESSLY ACKNOWLEDGES THAT THE DATA CONTAIN SOME NONCONFORMITIES, DEFECTS, OR ERRORS. ESRI DOES NOT WARRANT THAT THE DATA WILL MEET LICENSEE'S NEEDS OR EXPECTATIONS; THAT THE USE OF THE DATA WILL BE UNINTERRUPTED; OR THAT ALL NONCONFORMITIES, DEFECTS, OR ERRORS CAN OR WILL BE CORRECTED. ESRI IS NOT INVITING RELIANCE ON THESE DATA, AND THE LICENSEE SHOULD ALWAYS VERIFY ACTUAL DATA.

EXCEPT FOR THE LIMITED WARRANTY SET FORTH ABOVE, THE DATA AND RELATED MATERIALS CONTAINED THEREIN ARE PROVIDED "AS IS," WITHOUT WARRANTY OF ANY KIND, EITHER EXPRESS OR IMPLIED, INCLUDING, BUT NOT LIMITED TO, THE IMPLIED WARRANTIES OF MERCHANTABILITY AND FITNESS FOR A PARTICULAR PURPOSE.

Exclusive Remedy and Limitation of Liability: The entire liability of ESRI or its licensor/licensors and Licensee's exclusive remedy shall be to terminate the Agreement upon Licensee returning the Data and Related Materials to ESRI with a copy of Licensee's invoice/receipt and ESRI returning the license fees paid to Licensee.

IN NO EVENT SHALL ESRI AND/OR ITS LICENSOR/LICENSORS BE LIABLE FOR COSTS OF PROCUREMENT OF SUBSTITUTE GOODS OR SERVICES; LOST PROFITS, LOST SALES, OR BUSINESS EXPENDITURES, INVESTMENTS, OR COMMITMENTS IN CONNECTION WITH ANY BUSINESS; LOSS OF ANY GOODWILL; OR FOR ANY INDIRECT, SPECIAL, INCIDENTAL, EXEMPLARY, OR CONSEQUENTIAL DAMAGES ARISING OUT OF THIS AGREEMENT OR USE OF THE DATA AND RELATED MATERIALS, HOWEVER CAUSED, ON ANY THEORY OF LIABILITY, AND WHETHER OR NOT ESRI OR ITS LICENSOR/LICENSORS HAVE BEEN ADVISED OF THE POSSIBILITY OF SUCH DAMAGE. THESE LIMITATIONS SHALL APPLY NOTWITHSTANDING ANY FAILURE OF ESSENTIAL PURPOSE OF ANY EXCLUSIVE REMEDY.

Third Party Beneficiary: ESRI's licensor/licensors has (have) authorized ESRI to (sub) distribute and (sub)license its (their) data as incorporated into the Data and Related Materials. As an intended third party beneficiary to this Agreement, the ESRI licensor/licensors is (are) entitled to directly enforce, in its own name, the rights and obligations undertaken by the Licensee and to seek all legal and equitable remedies as are afforded to ESRI.

Q: How should I attribute the sample data provided on ESRI Data & Maps disks if I use it as prescribed above?

A: In the event that the data vendor/vendors has (have) granted the end user permission to redistribute the geodata, please use proper proprietary or copyright attributions for the various data vendor/vendors and provide the associated metadata file(s) with the geodata. In compliance with FGDC metadata standards, ESRI has attempted to practice proper metadata methodologies by providing any data source information, descriptions, and filenames to assist in this effort.

Related Topics

What is ESRI Data and Maps?

What's new in ESRI Data and Maps in ArcGIS 10

Copyright © 1995-2011 Esri. All rights reserved.

2/7/2011

URL: http://help.arcgis.com/en/arcgisdesktop/10.0/help/001z/001z00000003000000.htm
